# Bioproduction of cerium-bearing magnetite and application to improve carbon-black supported platinum catalysts

**DOI:** 10.1186/s12951-024-02464-x

**Published:** 2024-04-24

**Authors:** Jinxin Xie, Ziyu Zhao, Victoria S. Coker, Brian O’Driscoll, Rongsheng Cai, Sarah J. Haigh, Stuart M. Holmes, Jonathan R. Lloyd

**Affiliations:** 1 Department of Earth and Environmental Sciences, The University of Manchester, Manchester, UK; 2 Department of Chemical Engineering, The University of Manchester, Manchester, UK; 3 Department of Earth and Environmental Sciences, The University of Ottawa, Ottawa, Canada; 4 Department of Materials, The University of Manchester, Manchester, UK

**Keywords:** Fe(III) reduction, *Geobacter sulfurreducens*, Cerium, Microbial redox cycling, Mineral transformation, Platinum catalysts improvement, Oxygen reduction reactions

## Abstract

**Background:**

Biogeochemical processing of metals including the fabrication of novel nanomaterials from metal contaminated waste streams by microbial cells is an area of intense interest in the environmental sciences.

**Results:**

Here we focus on the fate of Ce during the microbial reduction of a suite of Ce-bearing ferrihydrites with between 0.2 and 4.2 mol% Ce. Cerium K-edge X-ray absorption near edge structure (XANES) analyses showed that trivalent and tetravalent cerium co-existed, with a higher proportion of tetravalent cerium observed with increasing Ce-bearing of the ferrihydrite. The subsurface metal-reducing bacterium *Geobacter sulfurreducens* was used to bioreduce Ce-bearing ferrihydrite, and with 0.2 mol% and 0.5 mol% Ce, an Fe(II)-bearing mineral, magnetite (Fe(II)(III)_2_O_4_), formed alongside a small amount of goethite (FeOOH). At higher Ce-doping (1.4 mol% and 4.2 mol%) Fe(III) bioreduction was inhibited and goethite dominated the final products. During microbial Fe(III) reduction Ce was not released to solution, suggesting Ce remained associated with the Fe minerals during redox cycling, even at high Ce loadings. In addition, Fe *L*_2,3_ X-ray magnetic circular dichroism (XMCD) analyses suggested that Ce partially incorporated into the Fe(III) crystallographic sites in the magnetite. The use of Ce-bearing biomagnetite prepared in this study was tested for hydrogen fuel cell catalyst applications. Platinum/carbon black electrodes were fabricated, containing 10% biomagnetite with 0.2 mol% Ce in the catalyst. The addition of bioreduced Ce-magnetite improved the electrode durability when compared to a normal Pt/CB catalyst.

**Conclusion:**

Different concentrations of Ce can inhibit the bioreduction of Fe(III) minerals, resulting in the formation of different bioreduction products. Bioprocessing of Fe-minerals to form Ce-containing magnetite (potentially from waste sources) offers a sustainable route to the production of fuel cell catalysts with improved performance.

**Graphical Abstract:**

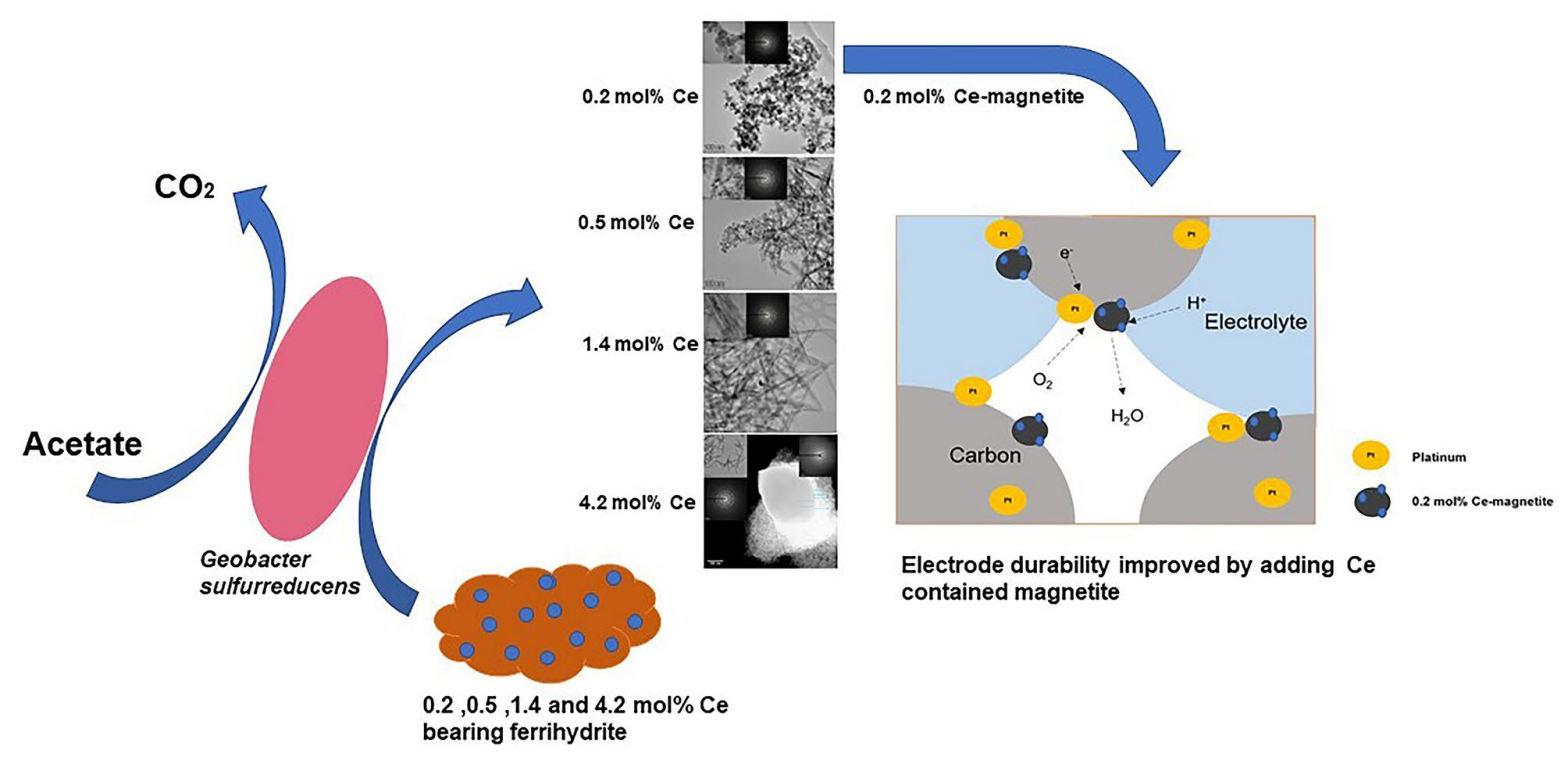

**Supplementary Information:**

The online version contains supplementary material available at 10.1186/s12951-024-02464-x.

## Introduction

Cerium (Ce) is the most abundant of the rare earth elements (REE) in the Earth’s crust, which comprise approximately 0.0046% of the Earth’s crust by weight [1, 2]. Cerium oxide is widely used in anthropogenic applications including as a polishing material, catalyst, ultraviolet absorber, fuel cell electrolyte and automobile exhaust absorber [2–5]. However, the mining and beneficiation of raw REE can cause serious environmental impacts. Indeed Liu (2016) highlighted the paradox that some rare earth elements products are being used to build a clean, smart, low-carbon and climate-resilient future while simultaneously causing significant environmental damage [6]. For example, in China, REE mining and extraction have caused water pollution of the Pearl River Basin, which has severely affected local agriculture and fisheries [6, 7]. Moreover, in the region of Baotou City, where there are large REE reserves, cancer cases have been reported in villages and attributed to buried radioactive REE mining wastes [6].

Unlike most of the other REE that exist in only the trivalent state, Ce(III) may be converted to tetravalent cerium (Ce(IV)) under oxidizing conditions in surface environments [8]. Ce(III) oxidation to Ce(IV) in the soil environment is closely related to dissolution/precipitation processes during weathering [8]. In addition, Ce(IV) is more readily adsorbed to bacteria or soils in natural environments than other trivalent REE [9]. Although Ce can exist in two oxidation states in the environment, most work on microbe-Ce interactions has focused on the sorption of Ce(III) to biomass (biosorption) [10]. For example Anders (2003) carried out REE biosorption experiments involving a range of microorganisms, which showed that the sorption capacity of bacteria varies greatly (2 to 1000 µmol/g biomass), with carboxylic or phosphate groups in the cell wall implicated in the sorption of REE cations [11]. Ohnuki et al. (2015) conducted experiments to compare the sorption of Ce(III) to synthetic Mn(IV) oxides and the soil bacterium *Pseudomonas fluorescens* [12]. Both Mn(IV) oxides and *Pseudomonas fluorescens* were shown to have the ability to remove Ce(III) ions from solution, with subsequent oxidation of Ce(III) to Ce(IV) associated with Mn(IV) oxides but not the bacterial cells. Ohnuki et al. (2015) also showed that the adsorbed Ce(III) on a bacterial cell reacted with P released from the cell to form nano Ce(III)-phosphate, and that these nano-particles inhibited oxidation of Ce(III) to Ce(IV) [12]. Furthermore, Ce-containing materials can have negative impacts on some microbial processes. This is illustrated by the work of Garcia (2012) who conducted experiments to investigate the effect of cerium oxide (CeO_2_) on a mixed wastewater treatment microbial community (including heterotrophic and ammonia-oxidizing bacteria) Here the addition of cerium oxide nanoparticles to the wastewater caused nearly 100% reduction in biogas production [13].

Dissimilatory metal-reducing bacteria (DMRB) can conserve energy for growth by coupling the oxidation of organic matter to the reduction of oxidized metals and metalloids, often altering their solubility [14]. For example, under anaerobic conditions, a wide range of metals such as Cu(II), U(VI), Pd(II), Ag(I) and Au(III) are bioreduced and can be precipitated as extracellular or intracellular metallic nanoparticles via these respiratory processes [14–18]. Also through the process of dissimilatory metal reduction, some minerals can be transformed into new phases; for example, *Shewanella oneidensis* can reduce Fe(III) in smectite, resulting in a structural change in the smectite and the formation of illite [19]. In addition, bacteria such as *Geobacter sulfurreducens* and *S. oneidensis*, can also reduce poorly crystalline Fe(III) minerals using organic matter as an electron donor, producing Fe(II)-bearing minerals such as magnetite, siderite (FeCO_3_) or vivianite (Fe_3_(PO_4_)_2_ ·8H_2_O) [14, 20, 21]. During the latter process, Fe(III) in the ferrihydrite is initially reduced to generate Fe(II) ions, which then combine with the Fe(III) mineral to form magnetite [22–25]. Previous work has studied the fate of metal ions associated with Fe(III)-oxyhydroxides (which have a high surface area and high sorption capabilities) during the process of dissimilatory metal reduction, with studies showing that contaminant metals such as Ni, Cr and Co can be incorporated into secondary minerals, such as the inverse spinel structure of magnetite, altering the physical/chemical properties of the mineral [26–28]. In contrast, the presence of other metals is known to alter the nature of the secondary minerals produced by bioreduction processes, such as high As content which can favour the formation of goethite from schwertmannite [22]. However, the impact of Ce on Fe(III) mineral bioreduction, as well as the associated final products, remains poorly understood. Iron minerals such as ferrihydrite, hematite, magnetite, and goethite are ubiquitous in the natural environment, and a high surface area and therefore high metal adsorption capacity [29]. REEs including cerium (Ce^3+^ and Ce^4+^ ions) can adsorb onto the surfaces of a range of iron minerals [29, 30], and therefore exhibit a close association with Fe phases in the environment [31–33]. Studies on the geochemistry of cerium associated with iron minerals are mainly limited to adsorption processes [10], despite the tight coupling of Fe and Ce in environmental systems, and the potential for redox cycling of Ce-bearing Fe minerals.

This study focuses on the bioreduction of Ce-bearing ferrihydrite by *G. sulfurreducens* to identify the fate of Ce species in Fe-rich environmental systems undergoing redox transformations. It also explores the feasibility of biosynthesising Ce-bearing magnetite, which could offer a new route for the synthesis of a potent catalyst for fuel cell electrodes [34, 35]. Experiments with different initial cerium concentrations were used to determine whether the REE influences the bioreduction of ferrihydrite, including exploring the final structure of any Fe(II)-bearing minerals formed, including biomagnetite. The relevance of our results to the environmental fate of REE, and the potential to harness this system for REE bioprocessing to produce novel Fe-based mineral nanoparticles, are discussed. In addition, carbon-black-supported platinum and bioreduced Fe/Ce-oxide catalyst are shown to offer a better durability with the use of less Pt when compared to commercially available alternatives.

## Materials and methods

### Cultivation of *Geobacter sulfurreducens*

An anaerobic bacterial growth medium (pH 7.0) containing an electron donor (25 mM sodium acetate) and electron acceptor (40 mM sodium fumarate) was prepared as described previously [36] and decanted to 100 ml serum bottles, then flushed with 80:20 mix of N_2_:CO_2_ for 20 min to remove oxygen and sealed with butyl rubber stoppers. The bottles were then autoclaved for 20 min at 126ºC and stored in the dark before use. A 10 ml aliquot of a fresh stationary phase culture of *G. sulfurreducens* was added to the bottles (which contained 90 ml anaerobic growth medium). The bottles were incubated at 30ºC until the cultures had reached late exponential phase and were then harvested by centrifugation at 5000 *g* and 4 ºC for 20 min. The cells were washed twice in sodium bicarbonate buffer (30 mM; pH 7) under an 80:20 mix of N_2_:CO_2_.

### Synthesis of Ce-bearing ferrihydrite

Four different Ce-bearing ferrihydrite preparations were synthesised (0.5% 1% 2% and 5% Ce mol%) from ferric chloride (FeCl_3_) and cerium chloride (CeCl_3_) stock solutions. The solutions were hydrolysed and precipitated by adding 10 M NaOH solution to a final pH 6.8-7.0, and the suspension was continually shaken for 1 h maintaining a constant pH value. The precipitate was then centrifuged at 5000 *g* at room temperature to separate the solid precipitate from the supernatant; the latter was discarded. An additional five washes were performed with deionized water to remove excess chloride ions, and the solid stored as a suspension in deionised water at 4^o^C. Inductively Coupled Plasma Atomic Emission Spectroscopy (ICP-AES) was used to measure the concentration of total Fe and Ce in the samples, and precipitates were also dried in air and powdered for analysis by X-ray diffraction (XRD).

### Microbial reduction of Ce-bearing ferrihydrite

Washed suspensions of *G. sulfurreducens* were added to a sodium bicarbonate solution (30 mM; pH7), containing Ce-bearing ferrihydrite (10 mmoles liter^-1^ slurry) and sodium acetate (10 mM) and incubated under an N_2_ and CO_2_ (80:20) headspace at 30ºC. Biomass loadings were equivalent to a final OD_600_ value of 0.4 (equivalent to 0.12 mg/mL biomass dry weight). Sample treatments were incubated in triplicate for each group to ensure reproducibility of experimental results. To measure the bioavailable Fe(II) generated, a ferrozine assay was used to monitor the production of Fe(II) over time [20, 37].

### Geochemical and mineralogical analysis

Inductively coupled plasma-atomic emission spectrometry (ICP-AES) was used to measure the concentration of iron and cerium in solution using a Perkin Elmer Optima 5300 dual view instrument. Starting materials (Ce bearing ferrihydrite) were processed by taking 0.1 ml of mineral slurry, which was digested in 4.9 ml 37% HCl for 30 min, filtered (0.22 μm) and then 0.1 ml of the digest added to 9.9 ml 2% HNO_3_ for ICP-AES to quantify Ce and Fe in starting materials.

ICP-AES was also used to detect whether cerium was released into solution during bioreduction. An aliquot (1 ml) of slurry was taken from the microbial incubations and centrifuged at 16,162*g* for 10 min, then 0.1 ml of the supernatant was added to 9.9 ml 2% HNO_3_ for ICP-AES analysis. The sampling points were the same as for the ferrozine assay sampling points.

A Bruker D8 system, operating at 40 kV/40 mA, with Cu Kα1 radiation (λ = 1.5406) was used for X-ray diffraction (XRD) analyses of solid samples. The angle of incidence (θ) was varied to change the diffraction angles (2θ) between 5° and 70°, with a step size of 0.02°, and the sample was rotated 360° to ensure all diffraction conditions were met [26]. Samples were ground to a uniform fine powder and then mounted on glass slides with amyl acetate in an anaerobic cabinet. XRD data analysis (background subtraction and peak identification) was performed by Diffrac. EVA with reference to the International Centre for Diffraction Data (ICDD) Powder Diffraction Database.

X-ray absorption near edge structure (XANES) characterization of solid materials was performed at the Ce *L*_*3*_-edge of samples to determine Ce oxidation state. An aliquot (1 ml) of slurry was taken and centrifuged (14800*g*). The supernatant was then discarded, and the sediment resuspended in 1 ml deionised water (DIW). After washing twice, 0.5 ml of slurry was dried anaerobically overnight, and then put onto a layer of Kapton tape and mounted onto an aluminium sample holder. XANES data for the starting materials were collected at room temperature at the Ce *L*_3_-edge (≈ 5727 eV) on beamline B18 at the Diamond Light Source (UK). The Athena software package was used to process the XANES raw data.

XAS (X-ray absorption spectroscopy) and X-ray magnetic circular dichroism (XMCD) at the Fe *L*_*2,3*_-edge were collected at the Advanced Light Source (ALS; Berkeley, USA) to measure the relative occupation of the three Fe ion sites within the magnetite structure [16, 38]. XAS data were collected in total-electron yield (TEY) mode, which gives an effective probing depth of ∼4.5 nm [28]. At each energy point, the XAS were measured for the two opposite magnetisation directions set parallel and anti-parallel to the beam direction with a magnetic field strength of 0.6 T. XMCD spectra were made by taking the difference between two normalised XAS spectra from the different magnetisation directions [39]. XMCD data and standards were fitted using the Qfit software to the three main peaks in the Fe *L*_3_-edge XMCD of magnetite, which broadly correspond to the relative quantity of Fe^2+^ O_h_, Fe^3+^ T_d_ and Fe^3+^ O_h_. These data have been previously reported to have an error on each site of up to 2% [28]. Samples were dried anaerobically, and then ground to powders in an anaerobic cabinet before loading onto carbon tape.

Transmission electron microscopy (TEM) and scanning transmission electron microscopy (STEM), performed on a probe corrected Thermo Fisher Titan G2 80–200, were used to image the morphology and structure of the bioreduced products. The STEM was equipped with Energy Dispersive X–ray Spectroscopy (EDS) (0.7 srad solid angle) and a high angle annular dark field (HAADF) STEM detector. The convergence angle for STEM was 21 mrad and the HAADF data were collected with an inner angle of 55 mrad at 200 kV. The distribution of different elements (Fe, Ce, O) in the post reduction products were characterized by EDS mapping in STEM mode. Selected area electron diffraction (SAED) patterns were also collected using a Thermo Fisher Talos STEM on the selected areas. For STEM sample preparation, the post reduction minerals were washed three times anaerobically using DIW and then drop cast onto an amorphous carbon film coated copper TEM grid.

### Synthesis and testing of catalysts

Biomagnetite and 0.2 mol% Ce-bearing magnetite were prepared through the bioreduction of ferrihydrite or 0.2 mol% Ce-bearing ferrihydrite by *G. sulfurreducens*. Platinum/carbon black (Pt/CB) supported catalysts (60% Pt) were synthesized using the biomaterials by first sonicating 50 mg of CB in 5 mL deionised water for 30 min to yield a fully dispersed solution. Subsequently, 166.7 mg chloroplatinic acid hexahydrate (H_2_PtCl_6_·6H_2_O) and 12.5 mg 0.2 mol% Ce-bearing biomagnetite (or biomagnetite) were added to 20 mL ethylene glycol (EG) to achieve a final 1:5 ratio of Fe and Pt. The mixed solution was then combined with the CB dispersed solution, sonicated for 2 h, and stirred for 1 h. Afterwards, the uniformly dispersed solution was transferred into a Teflon-steel autoclave to carry out the hydrothermal reduction process at 120 ℃ for 24 h. After cooling to room temperature, the catalyst solution was filtered under vacuum and washed with ethanol and deionised water to remove all residues (e.g. chlorides and solvent). Finally, the catalyst was dried in a vacuum oven at 60 °C overnight and stored at room temperature to give biomagnetite-Pt/CB (Biomag-Pt/CB) and 0.2 mol% Ce biomagnetite-Pt/CB supported catalysts (0.2%Ce-Biomag-Pt/CB). Pt/CB was also synthesized by the same process using 200 mg H_2_PtCl_6_·6H_2_O and a modified polyol reduction method [40, 41] as a control electrode material.

Electrochemical surface area (ECSA) and oxygen reduction reaction (ORR) activities were measured by cyclic voltammetry (CV) and linear sweep voltammetry (LSV), respectively. A conventional three electrode system was used, with an electrolyte solution of 0.5 M H_2_SO_4_, the counter electrode (platinum wire) and the reference electrode (Ag/AgCl), whilst a glassy carbon rotating disk electrode was used as the working electrode covered by catalyst. 5 mg of catalyst was dispersed in a mixture of 0.95 ml ethanol and 0.05 ml Nafion solution (5 wt%), following sonication for 1 h. Samples of catalyst suspension (20 µL) were dropped on to the glassy carbon rotating disk electrode. CV measurements were performed under a nitrogen-saturated atmosphere with a potential scan rate of 50 mV s^-1^ from − 0.2 to 1.0 V, rotating at a rate of 1600 rpm [42]. LSV testing was performed under an oxygen-saturated atmosphere with a scan rate of 20 mV s^-1^ from 1.0 to 0 V [42, 43]. The accelerated stress test (AST) was used to estimate the durability of catalysts for oxygen reduction reaction under oxygen saturated 0.5M H_2_SO_4_ solution. CV was performed with a scan rate of 100 mV s^-1^ between 0.6 and 1.0 V to accelerate the degradation of catalysts. After 18,000 cycles, the ECSA and ORR activity were tested again to quantify the long-term stability and activity of the catalysts.

## Results and discussion

### Synthesis and characterisation of Ce-bearing ferrihydrite

Although ferrihydrite was prepared with different target concentrations of Ce (0.5, 1, 2 and 5 mol% Ce), some Ce did not co-precipitate with the ferrihydrite during production and therefore the final solid phases contained 0.2, 0.5, 1.4 and 4.2 mol% Ce, respectively. XRD analyses of Ce-bearing starting materials confirmed the formation of poorly crystalline ferrihydrite with broad peaks observed at 35^o^ and 62^o^ 2θ (Figure S1) [44]. No differences in XRD patterns were observed with differing concentrations of Ce, and notably there was no evidence for the formation of crystalline cerium(IV) oxide, even at the higher Ce loadings (although such products may have been below the detection limit of XRD).

**Fig. 1:**
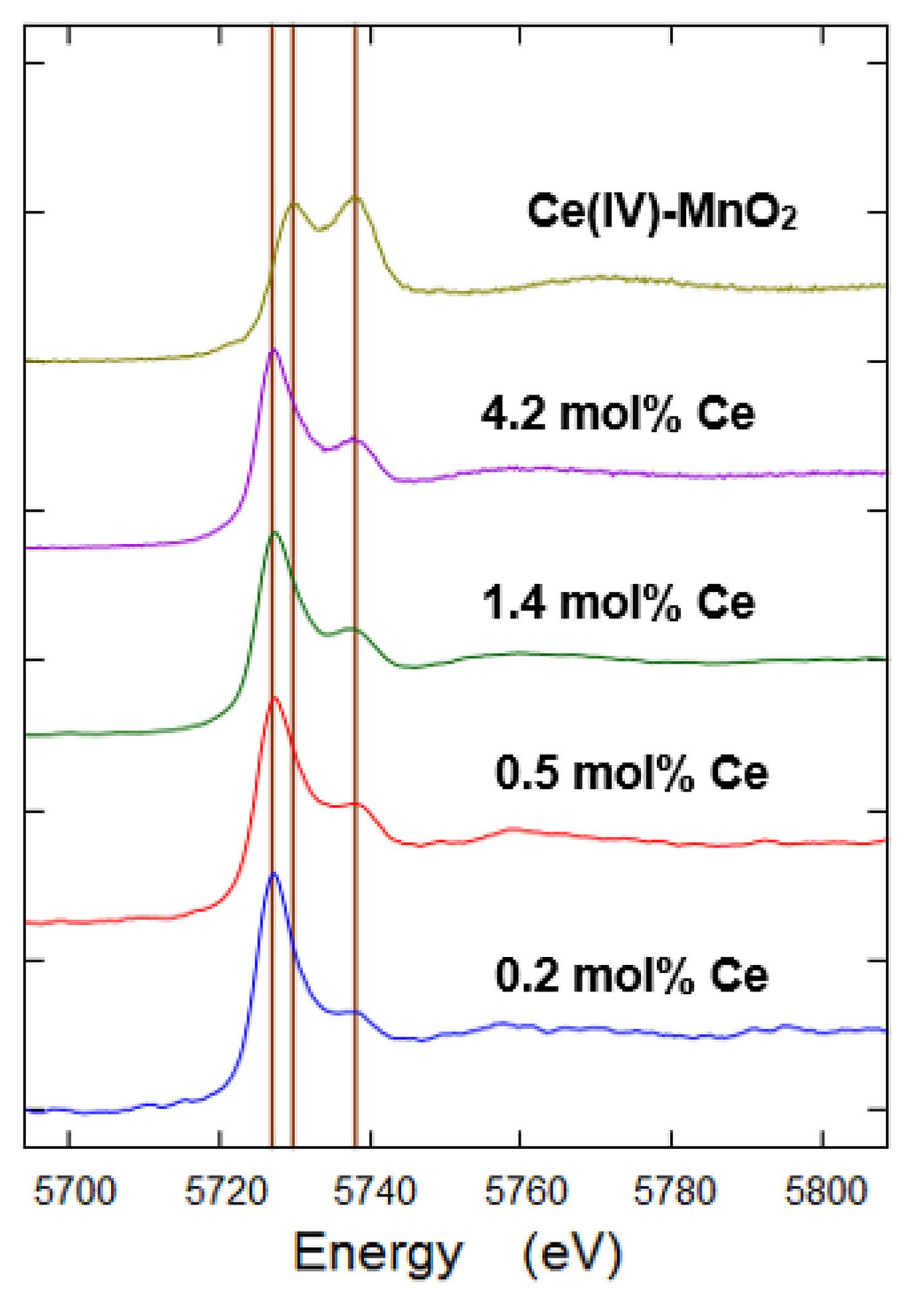
Normalized Ce K edge XANES of different concentrations of Ce-bearing ferrihydrite. Ce-MnO_2_ was used as Ce(IV) standard

Samples were analysed by Ce *K*-edge XANES, with a peak at 5727 eV characteristic of Ce(III), while peaks at 5730 eV and 5738 eV were specific for Ce(IV) [9, 45]. Ce *K*-edge XANES spectra of the ferrihydrite starting materials indicated the presence of both Ce(III) and Ce(IV), with progressively more Ce(IV) at higher loadings of cerium (Fig. 1) when compared to a Ce(IV)/MnO_2_ standard (apparent from an increase in peak height at 5738 eV relative to the Ce(III) peak at 5727 eV) [45]. Possible explanations for the presence of Ce(IV) could be that a portion of the Ce(III) was oxidized by air or Fe(III) during the precipitation of Ce-bearing ferrihydrite. In weakly acidic or alkaline solution, Ce(III) can be oxidized to Ce(IV), reaching 97% Ce(IV) hydroxide under optimal conditions [46]. Previous research has indicated that Ce(IV) dominates in high Fe content ferric deposits under oxidizing conditions and that the Ce(IV) is associated with newly formed Fe oxyhydroxides [32, 47–49]. However, recent studies [50] indicated that Ce(III) adsorbed to 2-line ferrihydrite cannot oxidize sorbed Ce(III) directly.

### Bioreduction of Ce-bearing ferrihydrite

**Fig. 2:**
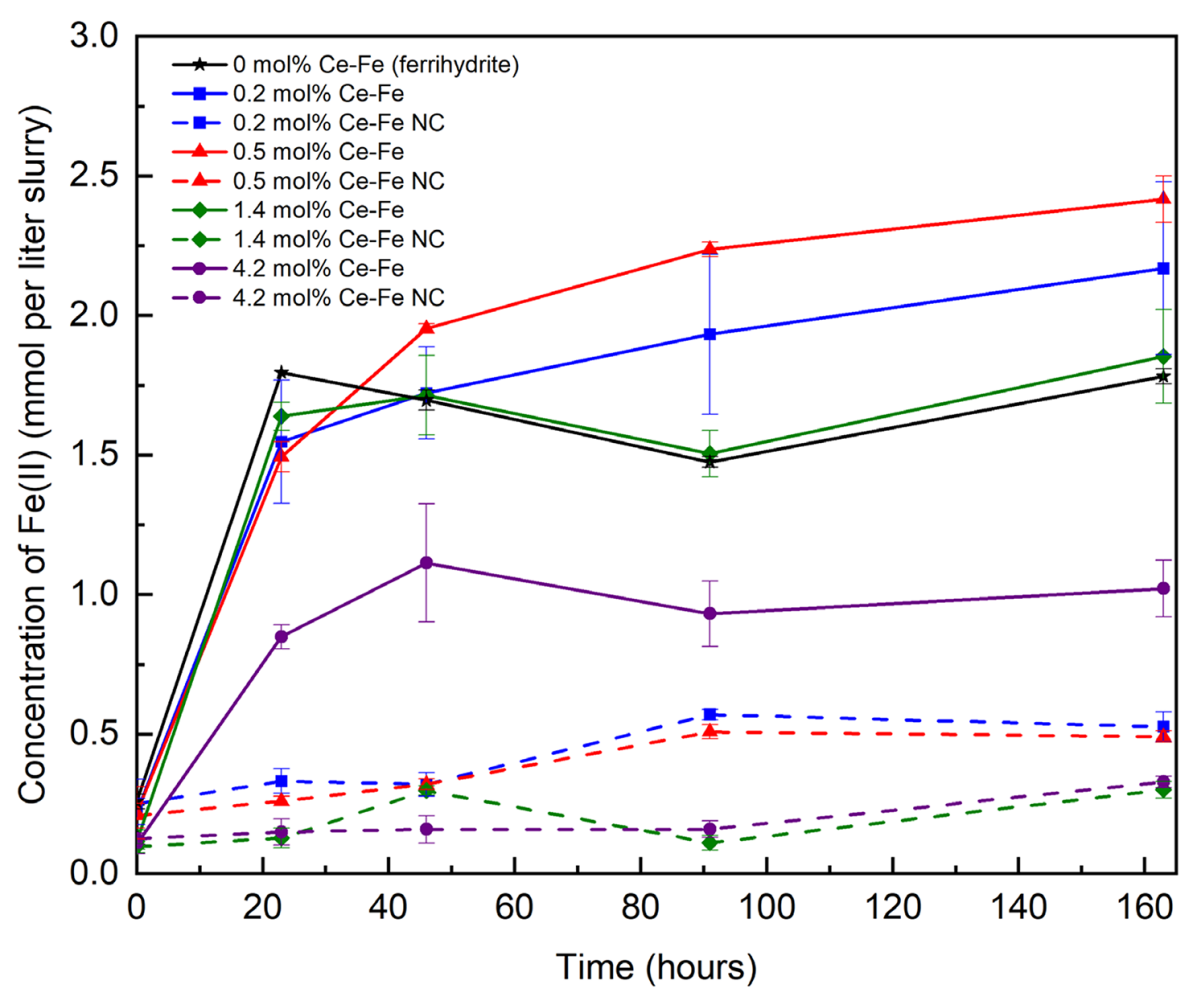
Changes in Fe (II) concentration in the 0.2 0.5 1.4 & 4.2 mol% Ce-bearing experiments (averages of triplicate incubations, each sample also measured in triplicate), NC refers to no cell control experiments

As expected microbial reduction of ferrihydrite resulted in an increase in Fe(II) over time, particularly in the first 24 h of incubation (Fig. 2). Experiments containing lower Ce-bearing ferrihydrite (0.2 mol%, 0.5 mol% and 1.4 mol% Ce) all behaved in a similar way in terms of the rate and extent of Fe(II) production for the first 24 h, giving between 1.5 and 1.8 mmoles liter^− 1^ Fe(II), only slightly lower than the results for Ce-free ferrihydrite (Fig. 2). There was a further gradual increase in Fe(II) for 0.2 mol% and 0.5 mol% Ce experiments up to 168 h reaching a maximum of around 2.4 mmoles liter^− 1^ slurry, whereas the 1.4 mol% Ce sample behaved as the Ce-free ferrihydrite, maintaining a similar lower Fe(II) value (1.6 to 1.8 mmoles liter^− 1^ slurry) from 24 h onwards. A black magnetic mineral formed in the 0.2 mol% and 0.5 mol% Ce bearing samples treatments, consistent with the conversion of ferrihydrite to magnetite [23], although this formed more quickly (after just 24 h) in the Ce free ferrihydrite incubations, compared to the Ce-bearing systems (∼ 48 h). In contrast, experiments containing 4.2 mol% Ce ferrihydrite reached a maximum of only 1.0 mmoles liter^− 1^ Fe(II) after 45 h and this remained constant until the end of the experiment at 168 h. For the 1.4 mol% and 4.2 mol% Ce samples, the end-point mineral at 168 h remained orange in colour and non-magnetic, despite the 1.4 mol% Ce following a similar Fe(II) evolution as the Ce-free experiment. These results suggest that Ce has less impact on the rate and extent of Fe(III) reduction at the lower Ce levels, but at higher loadings (1.4 mol% and 4.2 mol% Ce) the presence of the REE significantly impacted on secondary mineral formation and also inhibited Fe(II) production in the 4.2 mol% Ce experiment. Ce could inhibit Fe(III)-reduction for a number of potential reasons. For example the addition of Ce into the structure of the ferrihydrite [51] could impede electron flow [52–55] and therefore slow the reduction processes, or direct contact between the bacteria and Fe(III) surface sites could have been impeded due to the presence of Ce ions at the mineral surface. Negligible Fe(II) formed in cell-free control experiments (NC), confirming the importance of microbial electron transfer in the reduction processes reported here. During the bioreduction process, soluble Ce was monitored by ICP-AES at all time points but was not detected at any point (data not shown; the detection limits of Ce by ICP-AES is around 10 ppb), indicating that the microbial reduction of ferrihydrite by *G. sulfurreducens* does not mobilise quantifiable levels of Ce ions associated with the Fe mineral.

### Characterisation of secondary Fe minerals

**Fig. 3:**
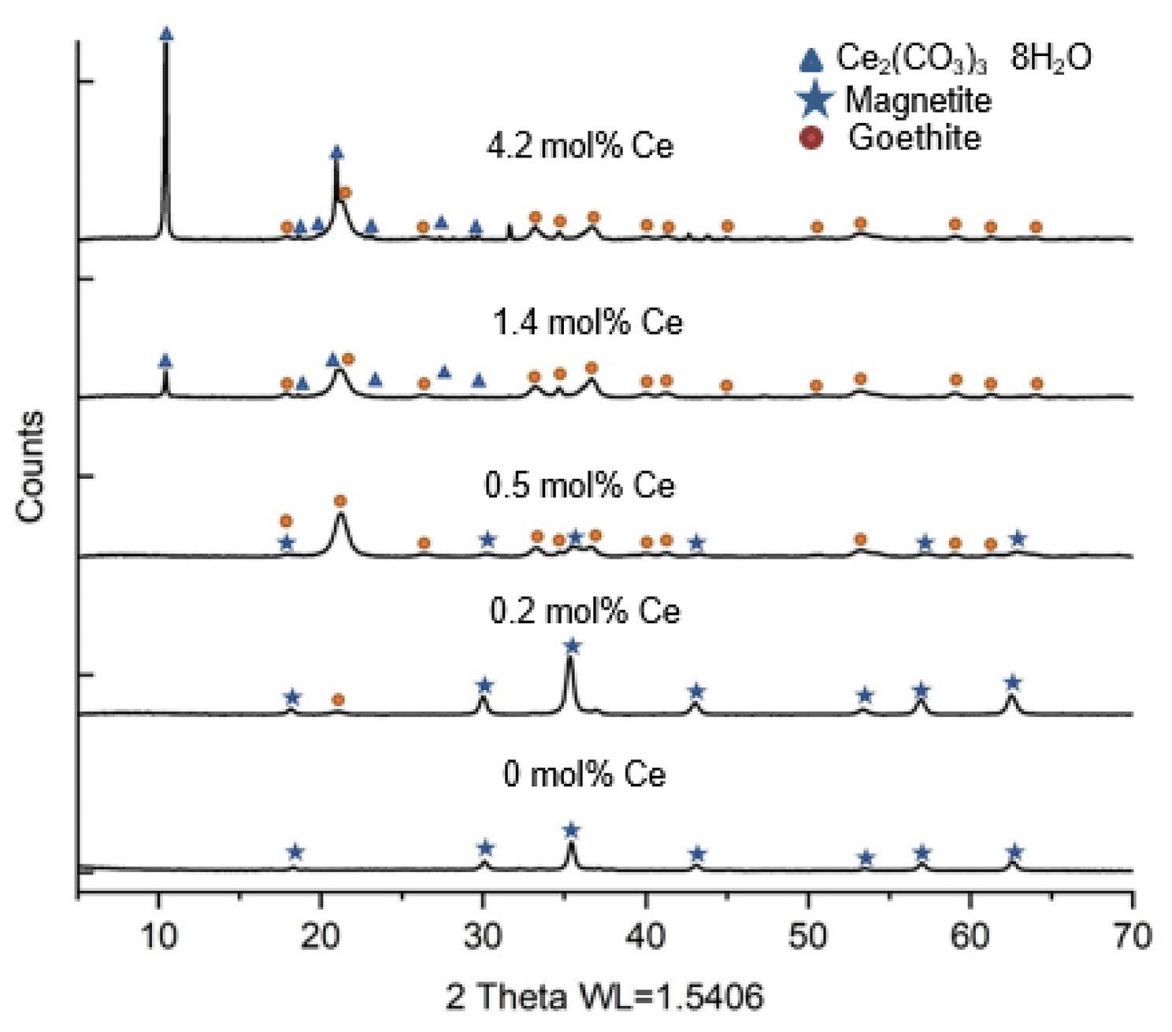
XRD traces of post reduction samples with different proportions of Ce (0,0.2, 0.5, 1.4 and 4.2 mol%)

XRD analyses of post-reduction biomineral products indicated that magnetite (PDF 00-019-0629) [56] was produced in the low Ce bearing experiments (0.2 and 0.5 mol%) (Fig. 3). However, the XRD spectra also contained peaks at 22, 34, 36, 54 and 57 2-theta in the 0.5 mol% Ce sample, suggesting the presence of a significant proportion of goethite (PDF 00-029-0713) [57] in addition to magnetite. For high Ce-bearing samples (1.4 mol% and 4.2 mol%), goethite was the only Fe-bearing mineral identified in the post reduction products, but cerium(III) carbonate octahydrate (PDF 00-038-0377) [58] was also observed. According to previous research, various factors, including pH, Fe(II) concentration, temperature, and the presence of electron shuttles, significantly influence the products of Fe(III) bioreduction [59–63]. Under the near-neutral conditions, Fe(II) ions can be a catalyst for the conversion of ferrihydrite into goethite [64]. However, at pH values greater than 7 and at higher Fe(II) concentrations, the dominant products tend to be Fe(II)-rich minerals such as magnetite, with the Fe(III) mineral goethite typically present as an intermediate product [55, 61]. In our experiments, the initial pH was around 7.5–8.3, and this pH was maintained throughout the bioreduction process. Ferrozine assays indicated that with increased Ce content, the formation of Fe(II) was inhibited, and magnetite only formed in low Ce samples, while goethite was the dominant product at higher (1.4 mol% and 4.2 mol%) Ce loadings. Ce carbonate was also detected at higher Ce loadings, and it is possible that during the bioreduction process some adsorbed Ce(III) combined with carbonate ions from the buffer. However, based on the ICP-AES data collected during the bioreduction process, the Ce concentrations in solution were always below detection limits (around 10 ppb), indicating that Ce carbonate formation would have been associated with the iron mineral surface.

The Ce content affected the crystallite size of the biomagnetite, with smaller particles found at higher Ce levels up to 0.5 mol%. In the 0, 0.2 and 0.5 mol% Ce-bearing samples the magnetite crystals were 37 nm, 20 nm and 11 nm, respectively (calculated from the XRD data using the Scherrer equation) [26, 65, 66]. Magnetite nanoparticles aggregate owing to the strong magnetic dipole-dipole interactions that occur between particles, coupled with an inherent high surface energy, which exceeds 100 dyn/cm [67, 68]. Previous research has indicated that increased rates of bioreduction (e.g. with higher biomass loadings) have been noted to correlate with decreasing biomagnetite nanoparticle size [59], but in this study, slower rates of Fe(III) reduction (in the presence of Ce) led to the production of smaller magnetite nanoparticles. This is likely due to the presence of Ce on the surface of the Fe(III) substrate, changing the surface properties of the substrate and limiting particle aggregation. This phenomenon has been noted in abiotic studies on the formation of Ce-bearing magnetite nanoparticles, where increased Ce content led to smaller nanoparticles [68]. The large crystallite size and needle-like morphology of goethite in the 1.4 and 4.2 mol% Ce samples precludes precise calculation of goethite crystallite size using the Scherrer equation.

**Fig. 4:**
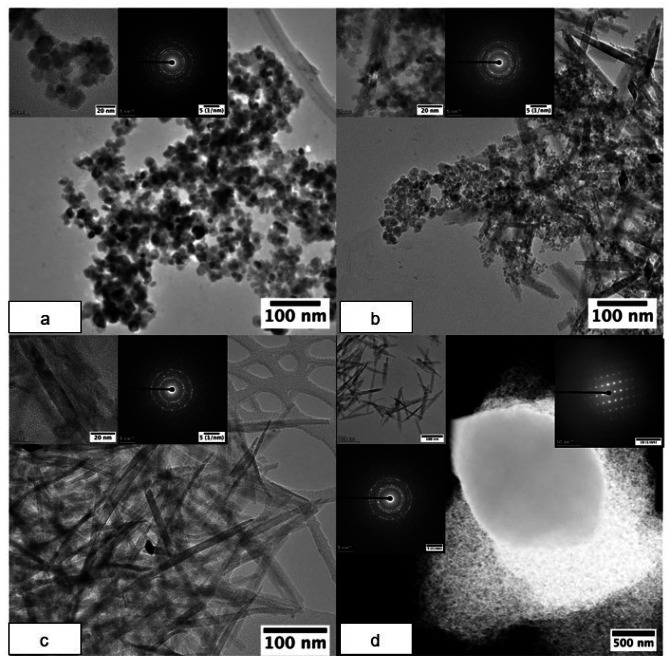
TEM images of (**a**) 0.2 mol%, (**b**) 0.5 mol%, (**c**) 1.4 mol% and (**d**) 4.2 mol% Ce-bearing post reduction products and corresponding TEM SAED results. Reflections (yellow rings) in sample A are all representative of magnetite (PDF 00-019-0629). Reflections (yellow rings) in B are representative of magnetite and goethite - the reflections of goethite have been marked with red dots to separate them from those of magnetite in the b) 1 mol% sample. Reflections (yellow rings) in sample C and D represent goethite (PDF 00-029-0713)

TEM images and SAED results (Fig. 4) were consistent with XRD data, with small, spherical magnetite nanoparticles only present in the low Ce-bearing samples (0.2 and 0.5 mol%), while large, needle-like goethite crystals were visible in the higher Ce-bearing samples. The products of the 0.5 mol% Ce-sample contained both rounded particulates and needle-like products, and the SAED results show the presence of both magnetite reflections and goethite (212) and (200) reflections (marked by red spots and highlighted in Fig. 4), consistent with the identification of both minerals in the 0.5 mol% sample by XRD. According to the TEM images (Fig. 4), the sizes of the magnetite nanoparticles formed in the 0.2 and 0.5 mol% Ce-bearing samples were 19.2 nm (standard deviation, SD = 3.1 nm, 50 particles) and 9.6 nm (SD = 2.4 nm, 30 particles), respectively, in excellent agreement with the crystallite sizes of ∼ 20 nm and 11 nm calculated from the XRD results. Goethite needles were visible in the bioreduction products of the 0.5, 1.4 and 4.2 mol% Ce-bearing samples. The length of the goethite needles was 140.8 nm (SD = 11.8 nm, 10 particles) and 150.7 nm (SD = 16.2 nm, 15 particles) in the 1.4 and 4.2 mol% Ce samples, but slightly smaller in the 0.5 mol% Ce sample, at about 95.5 nm (SD = 33.3 nm, 10 samples). Relatively large (micrometer diameter) Ce-bearing single crystal particles were present in the 4.2 mol% Ce-bearing sample. These might be expected to correspond to the cerium(III) carbonate octahydrate indicated in the XRD results, but STEM EDS elemental mapping and ESEM-EDS results (Figure S2 and S3) showed that these crystals contained Fe in addition to Ce, C and O. This suggests a carbonate compound with a similar crystal structure to cerium(III) carbonate octahydrate but containing Fe. The STEM EDS mapping results (Figure S2) also indicated that Ce was closely spatially associated with all Fe-bearing minerals in the bioreduction products. Unlike XRD, goethite was not identified with TEM in the 0.2 mol% Ce-bearing samples, or cerium(III) carbonate octahydrate in the 1.4 mol% Ce-bearing samples, possibly due to these crystals being relatively sparsely distributed, and the TEM analyzing a relatively small volume of material.

X-twere collected at the Fe *L*_*2,3*_-edge to investigate the structure of the magnetic biominerals that were formed (Figure S4). Fitting the spectra using calculated data for the three potential Fe environments within magnetite (Fe^2+^ O_h_, Fe^3+^ T_d_ and Fe^3+^ O_h_) can provide information on the oxidation state and site occupancies of the Fe cations.

Stoichiometric magnetite has an Fe occupancy of 1:1:1 in each of the Fe^2+^O_h_: Fe^3+^T_d_: Fe^3+^O_h_ sites [38], similar to the Fe occupancies measured in the biogenic magnetite produced from the pure ferrihydrite in the current study (Table 1). However, for 0.2 and 0.5 mol% Ce-bearing samples, the occupancy of Fe(III) decreased in both the T_d_ and O_h_ sites when compared to the standard biogenic magnetite. In general, the Fe(II) to Fe(III) ratio in the Ce-magnetite samples were much higher than that of the biogenic magnetite, and the ratio in 0.5 mol% Ce-bearing samples was highest. In addition, the proportion of Fe(III) O_h_ in the Ce-bearing magnetic samples (0.2 and 0.5 mol% Ce) were both lower than that in the biomagnetite, and the 0.5 mol% Ce-bearing sample had the least amount of Fe(III) O_h_ of all samples, suggesting that with increasing Ce, the content of Fe(III) O_h_ in the biomagnetite decreased. These data may indicate that Ce was partially incorporated into the Fe(III) sites of the magnetite and replaced some Fe(III) in order to maintain charge balance [69, 70].

**Table 1:** Fe site occupancies and the ratio of T_d_/O_h_ and Fe(II)/Fe(III) for all samples showing biomagnetite as a product, calculated from XMCD in Fig. S4

sample name	d^6^ Fe(II) O_h_	d^5^ Fe(III) T_d_	d^5^ Fe(III) O_h_	total	T_d_/O_h_	Fe(II)/Fe(III)
0% Ce ferrihydrite	1.05	1.05	0.91	3.01	0.54	0.54
0.2 mol% Ce	1.05	0.81	0.73	2.59	0.46	0.68
0.5 mol% Ce	1.05	0.80	0.62	2.47	0.48	0.74

### Application of biomagnetite and 0.2 mol% Ce-bearing magnetite in carbon black-supported platinum catalysts for oxygen reduction reactions

Hydrogen fuel cells are a promising future power source for portable electric devices and vehicles due to their inherent zero-carbon emissions and high power density [71, 72]. In a hydrogen proton exchange membrane fuel cell, hydrogen is oxidized at the anode and oxygen is reduced at the cathode. However, the oxygen reduction kinetics at the cathode are relatively slow compared to the hydrogen oxidation reaction at the anode, as the breaking of O-O bonds is challenging, and therefore efficient catalysts are necessary to improve the energy conversion efficiency of the whole system [73]. According to previous studies, pure platinum and platinum-based catalysts have the highest catalytic activity for oxygen reduction reactions (ORR) [73]. However, as a precious metal, platinum has a high market value, and sourcing non-noble metal catalysts in order to reduce the quantity of platinum required has become an important research focus [74, 75]. Cerium oxide (CeO_2_ and Ce_2_O_3_) and magnetite are both used as catalysts in fuel cell electrodes [76–78], and there is merit therefore in using an environmentally benign bioreduction process to form a Ce-bearing magnetite catalyst for potential applications in fuel cell electrodes [34, 35] .

According to XRD and TEM results, magnetite was detected in both 0.2 and 0.5 mol% Ce-bearing samples, although the 0.5 mol% Ce bearing sample contained high levels of goethite contamination and yielded poor electrochemical results in preliminary tests. Therefore, catalytic experiments focused on the 0.2 mol% Ce biomagnetite, with performance compared to a commercial carbon black supported platinum catalyst (Pt/CB). The biogenic material was incorporated into the Pt/CB catalyst, reducing the overall requirement for Pt, while a biomagnetite without Ce was also incorporated into Pt/CB as an additional control catalyst. Several sets of tests on all catalysts were processed, and overall trends (comparison of catalytic performance and durability) were consistent across these data sets.

Electrochemical characterization was conducted to assess the activity of the catalysts. Electrochemical surface areas (ECSA) of Pt/CB containing pure biomagnetite (Biomag-Pt/CB), 0.2 mol% Ce bearing magnetite (0.2%Ce-Biomag-Pt/CB) and the standard Pt/CB catalyst are shown in Figure S5 and Table 2 for comparison. 0.2%Ce-Biomag-Pt/CB showed the largest ECSA of 17.14 ${m}^{2}\cdot {g}^{-1}$, which was higher than that of Biomag-Pt/CB and Pt/CB, at 15.38${m}^{2}\cdot {g}^{-1}$ and 14.07${m}^{2}\cdot {g}^{-1}$ respectively. This result indicated that 0.2%Ce-Biomag-Pt/CB contained more active sites than Biomag-Pt/CB and Pt/CB, delivering a higher catalytic performance for the Ce-bearing material.

**Table 2:** Electrochemical characterization, including Electrochemical surface areas (ECSA), the onset potential (E_0_) half-wave potential (E_(1⁄2)_), diffusion-limiting current density (J), of catalysts before and after accelerated stress test (AST).

Catalysts	Before AST					After AST			
	ECSA (m^2^/g)	E_0_ (V)	E_1/2_ (V)	J (mA/cm^2^)		ECSA (m^2^/g)	E_0_ (V)	E_1/2_ (V)	J (mA/cm^2^)
0.2%Ce-Biomag-Pt/CB	17.14	0.85	0.64	-5.36		12.84	0.85	0.60	-5.03
Biomag-Pt/CB	15.38	0.85	0.64	-4.69		4.76	0.85	0.60	-4.30
Pt/CB	14.07	0.81	0.60	-5.40		4.73	0.76	0.50	-5.01

**Fig. 5:**
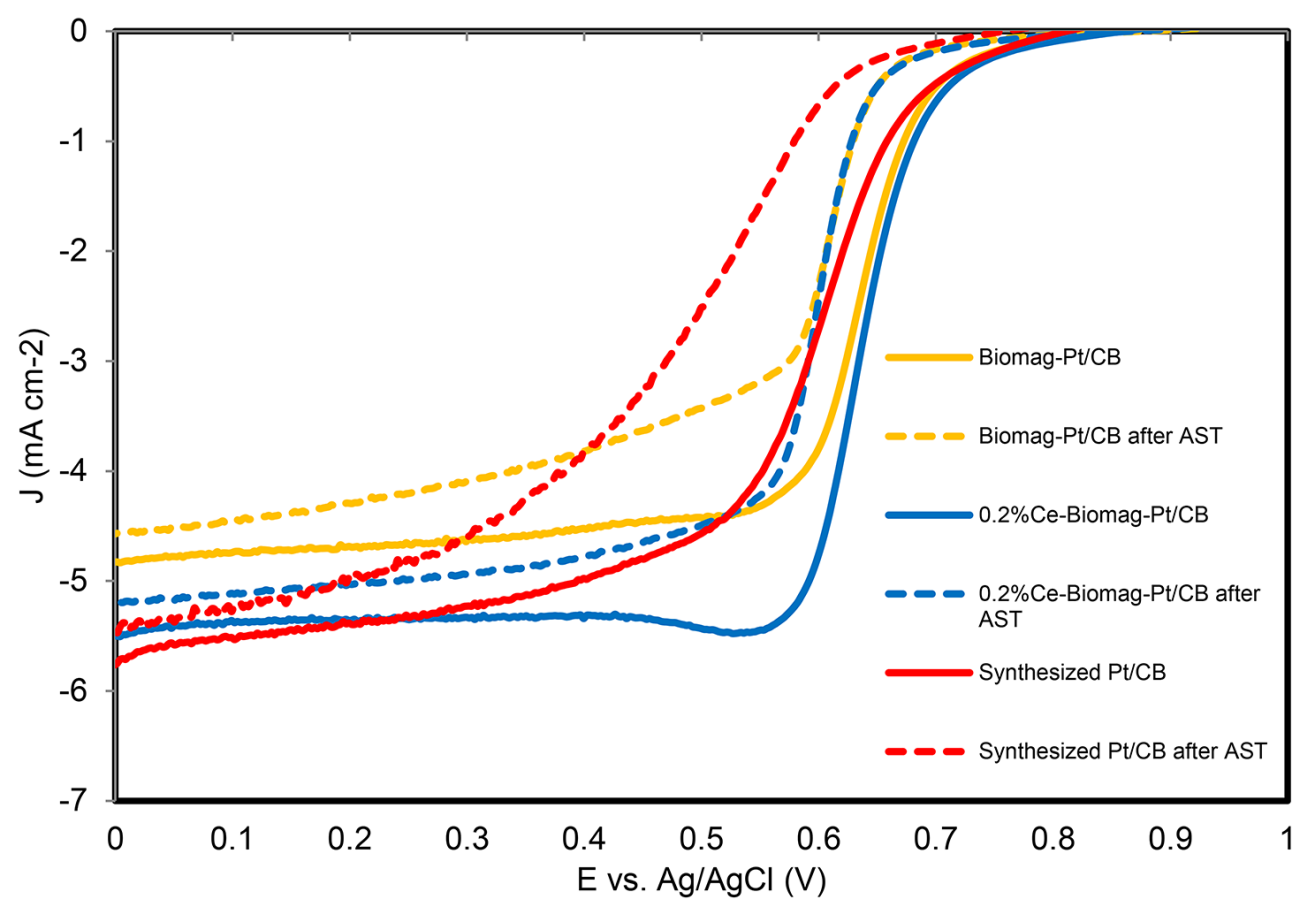
linear sweep voltammetry (LSV) before (solid lines) and after (dotted lines) 18,000 cycles of AST.

Linear sweep voltammetry (LSV) was also used to estimate the activities of all catalysts for the ORR under a rotating speed of 1600 rpm (Fig. 5). The diffusion-limiting current density of Pt/CB was similar to that of 0.2%Ce-Biomag-Pt/CB (approximately − 5.4 $mA\cdot {cm}^{-2}$), while the Biomag-Pt/CB sample performed less favourably with a diffusion-limiting current density of -4.30 $mA\cdot {cm}^{-2}$(see Table 2). The half-wave potential (${E}_{1/2}$) of 0.2%Ce-Biomag-Pt/CB was similar to Biomag-Pt/CB (0.64 V), which was 0.04 V higher than that of the Pt/CB (0.60 V). The results of the diffusion-limiting current density and half-wave potential measurements suggested that the 0.2 mol% Ce-magnetite improved the ORR activity slightly. In addition, the onset potential (${E}_{0}$) of Pt/CB was approximately 0.81 V, while the Biomag-Pt/CB and 0.2%Ce-Biomag-Pt/CB had higher onset potentials of 0.85 V. This indicated that the addition of biomagnetite and Ce-magnetite expedited the start of the oxygen reduction reaction. Overall, the Ce-magnetite supplemented Pt/CB catalyst showed a slight improvement in catalytic abilities compared to conventional Pt/CB catalysts and used 17% less Pt than the conventional catalyst, which would decrease the cost of production.

The performance of the Biomag-Pt/CB, 0.2%Ce-Biomag-Pt/CB and Pt/CB containing electrodes were also estimated after accelerated stress tests (AST) followed by the ECSA (Figure S6 and Table 2). The ECSA of Biomag-Pt/CB declined dramatically with 69.1% loss after 18,000 cycles of AST. There was a 66.4% loss of initial ECSA with the Pt/CB materials, while the ECSAs decreased by only 25.1% for the 0.2%Ce-Biomag-Pt/CB. (Figure S6). This demonstrated that the 0.2%Ce-Biomag-Pt/CB offered improved durability compared to either Biomag-Pt/CB or conventional Pt/CB.

A comparison of ORR activity before (solid lines) and after (dotted lines) 18,000 cycles AST was also carried out to confirm the durability of these catalysts (Fig. 5). Again the 0.2%Ce-Biomag-Pt/CB material appeared relatively stable with only a small decrease of half-wave potential between 0.64 V and 0.60 V, while significant decreases from 0.6 V to 0.5 V was evident for the Pt/CB material. These results show that the Ce-magnetite material substantially improved the stability of the Pt/CB catalyst. The diffusion-limiting current density of the 0.2%Ce-Biomag-Pt/CB electrode was reduced by only 6.2% (-5.36 to -5.03 $mA\cdot {cm}^{-2}$), compared to 7.2% and 8.3% losses with the Pt/CB and Biomag-Pt/CB materials, respectively (decreased to -5.01 $mA\cdot {cm}^{-2}$ and − 4.30 $mA\cdot {cm}^{-2}$). This also demonstrated the improved durability of 0.2%Ce-Biomag-Pt/CB compared to Pt/CB. According to the research of Masuda et al. (2012) [79], metallic Pt is a more suitable catalyst for ORR activity compared to Pt oxides. This suggests that the greater durability of the 0.2%Ce-Biomag-Pt/CB may be due to the presence of Ce^3+^ which could be oxidized to Ce^4+^, limiting the formation of Pt oxides ^[79]^.

In summary, an electrode containing 0.2%Ce-Biomag-Pt/CB possessed a higher ORR activity when compared to a standard Pt/CB electrode before AST and retained 93.8% of the diffusion-limiting current density after 18,000 cycles. Thus, the addition of Ce-bearing biomagnetite to a Pt/CB electrode can lead to improved electrode durability for catalytic ORR applications, which in turn will reduce operating costs due to the reduced requirement for Pt.

## Conclusion

In conclusion, when Ce was co-precipitated with ferrihydrite, it was successfully reduced by *G. sulfurreducens* to yield a Ce-bearing magnetite when the Ce concentration was relatively low, and at higher concentrations (1.4 mol% Ce content) microbial Fe(III) reduction was inhibited and products were dominated by goethite. The lack of soluble Ce suggested the Fe minerals had a high sorption capacity for Ce, which could have implications for natural and engineered systems for the treatment of REE containing waters. This work also opens up a route for the sustainable production of novel functional biomaterials for technological use via microbial revalorisation of waste streams (confirmed in principle for waste Fe(III) materials [16]). This is illustrated by the superior durability of Pt/CB fuel cell catalysts when supplemented with 0.2 mol% Ce-biomagnetite, which contained 17% less platinum compared to commercial Pt/CB. These proof of concept experiments point the way to novel bioprocessing options for REE materials, that could be further expanded and engineered for applications using a range of approaches including optimization of feedstocks and biological activity (the latter through strain selection and biological engineering approaches).

### Electronic supplementary material

Below is the link to the electronic supplementary material.

Supplementary Material 1

## Data Availability

The datasets used and/or analysed during the current study are available from the corresponding authors on reasonable request.
