# Supplementary material for: Bioproduction of cerium-bearing magnetite and application to improve carbon-black supported platinum catalysts
Source: J Nanobiotechnology. 2024 Apr 24;22:203. doi: 10.1186/s12951-024-02464-x (PMC11041677; doi:10.1186/s12951-024-02464-x)
Supplement: Supplementary file 1 — Supplementary Material 1 [file 12951_2024_2464_MOESM1_ESM.docx]

Bioproduction of cerium-bearing magnetite and application to improve carbon-black supported platinum catalysts

**Jinxin Xie*, Ziyu Zhao, Victoria S. Coker, Brian O'Driscoll, Rongsheng Cai,**

**Sarah J. Haigh, Stuart M. Holmes, Jonathan R. Lloyd***


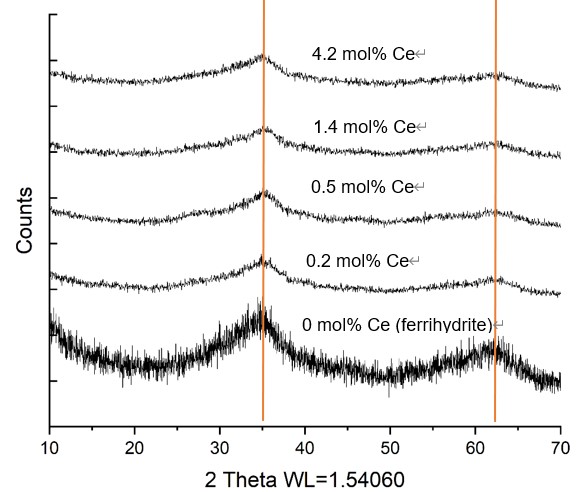


Figure S1 X-ray diffraction (XRD) results obtained from Ce-bearing ferrihydrite with different Ce concentrations (0, 0.2, 0.5, 1.4 and 4.2 mol%). Orange lines indicate reflections at 35 and 62 deg 2-theta , indicative of 2-line ferrihydrite[1]


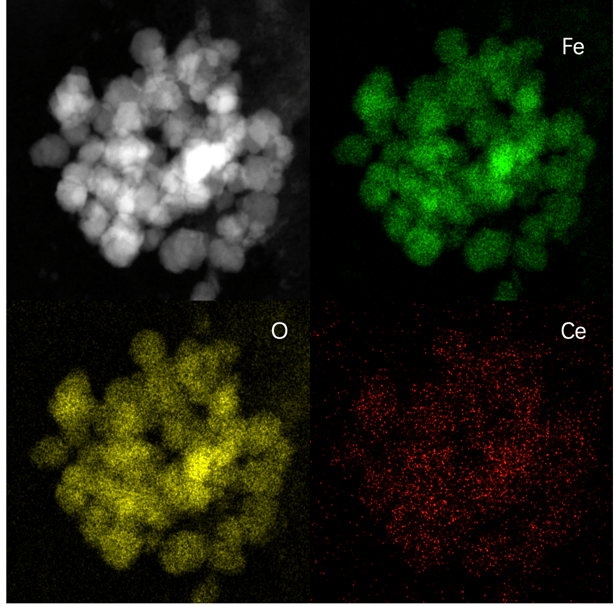

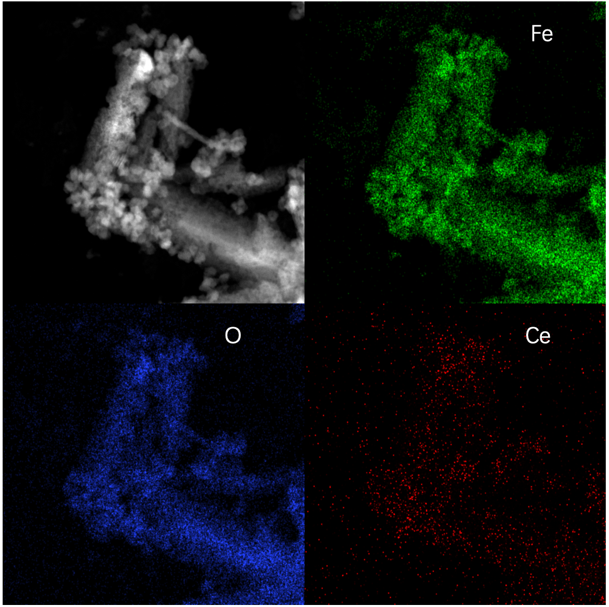

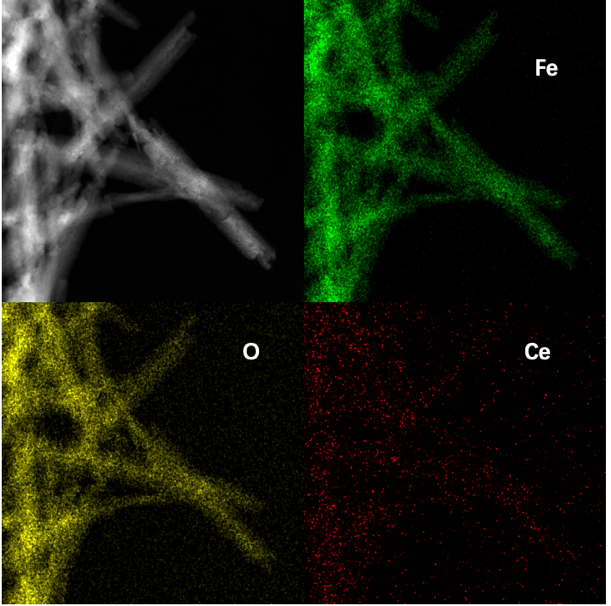

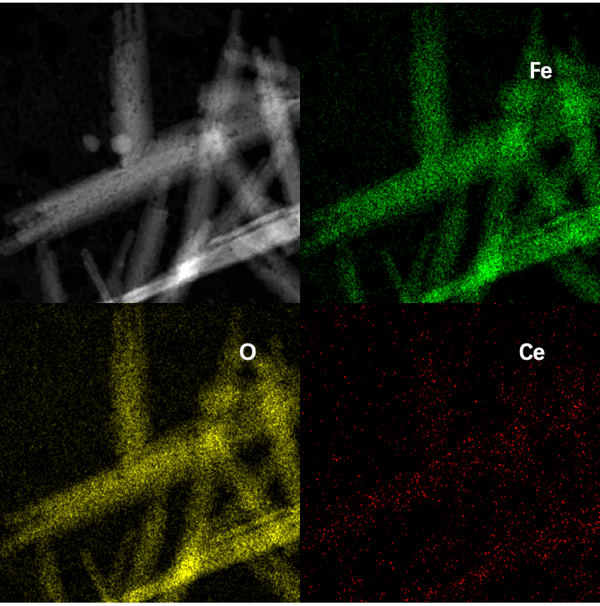


**C**

**D**

**B**

**A**


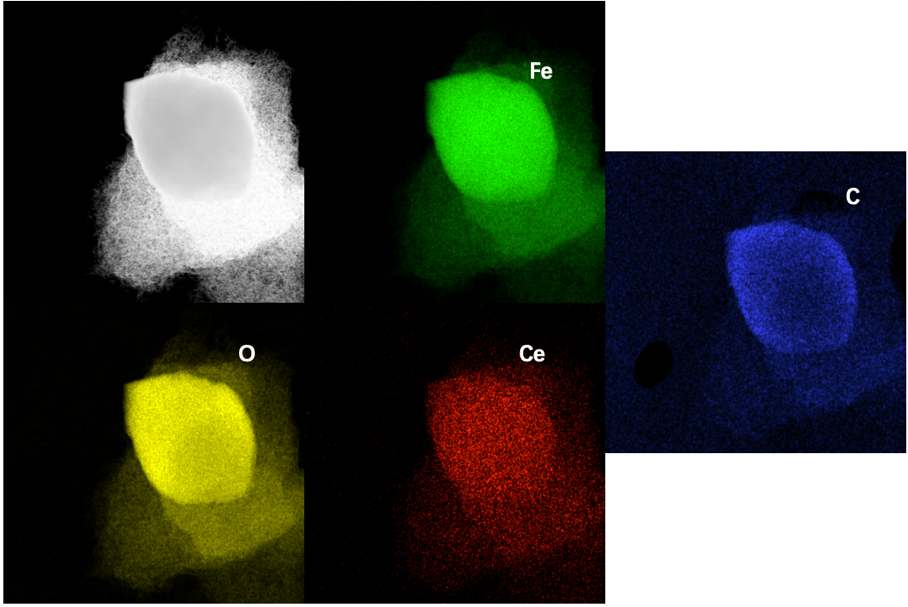


**E**

Figure S2: scanning transmission electron microscopy (STEM) high angle annular dark field (HAADF) and Energy Dispersive X–ray Spectroscopy (EDS) elemental mapping images of post reduction products for ferrihydrite containing .A) 0.2, B) 0.5, C) 1.2, D) and E) 4.2 mol% Ce

Fe, O, Ce and C are shown green, yellow, red and blue, respectively. A: The bioreduction product of 0.2% samples was magnetite. B: The bioreduction products of 0.5% samples were magnetite and goethite. C: The bioreduction product of 1.4% samples was goethite. For 4.2 mol% Ce samples there were two different minerals in the products D: Goethite and. E: carbonate mineral containing Fe and Ce. The apparently lower content of C and O in the micrometer sized particle in E is an artefact due to absorption of these light X-rays.








Figure S3: Environmental scanning electron microscope (ESEM) image of 5% Ce bearing samples bioreduction product and EDS results of selected needle shaped minerals


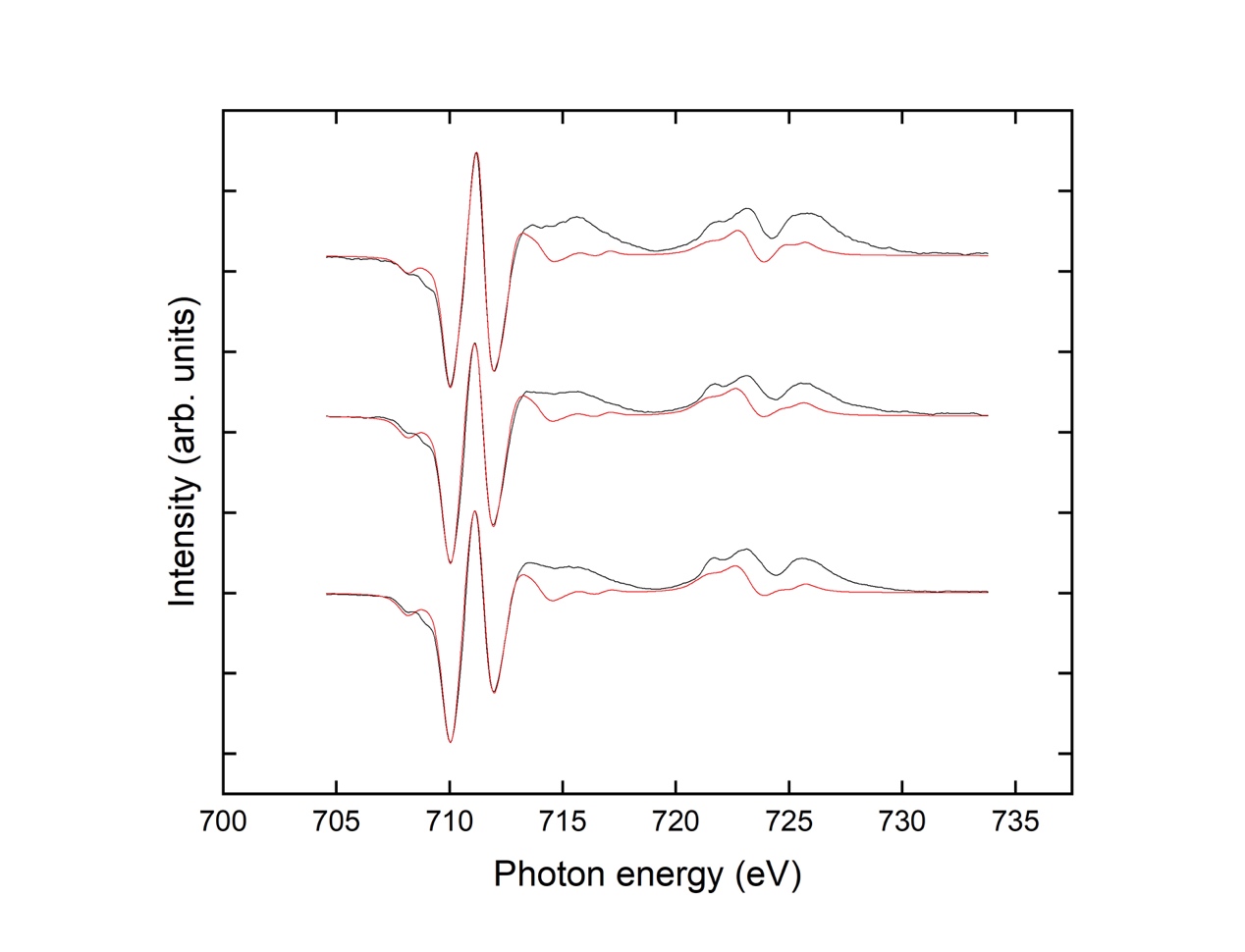


0.2 mol% Ce

0 mol% Ce

0.5 mol% Ce

Figure S4. Fe *L*_2,3_-edge X-ray magnetic circular dichroism (XMCD) spectra data (black line) and best fit (red line) calculated spectra for the 0, 0.2 and 0.5 mol% Ce magnetic bioreduction products.

Figure S5. cyclic voltammetry (CV) curves results of 0.2%Ce-Fe-Pt/CB, Fe-Pt/CB and Pt/CB.

**A**

**B**

**C**

Figure S6. Electrochemical surface area (ECSA) results of different catalyst materials before and after AST. A) 0.2%Ce-Fe-Pt/CB, B) Fe-Pt/CB and C) Pt/CB.

Reference:

1. Schwertmann U, Cornell RM: **Iron oxides in the laboratory: preparation and characterization**: John Wiley & Sons; 2008.
